# Moderate Effects of Hypoxic Training at Low and Supramaximal Intensities on Skeletal Muscle Metabolic Gene Expression in Mice

**DOI:** 10.3390/metabo13101103

**Published:** 2023-10-21

**Authors:** Svitlana Drozdovska, Nadège Zanou, Jessica Lavier, Lucia Mazzolai, Grégoire P. Millet, Maxime Pellegrin

**Affiliations:** 1 Institute of Sport Sciences, University of Lausanne, 1015 Lausanne, Switzerland; svitlana.drozdovska@unil.ch (S.D.); nadege.zanou@unil.ch (N.Z.); jessica.lavier@unil.ch (J.L.); 2 Biomedical Disciplines Department, Health, Physical Education and Tourism Faculty, National University of Ukraine on Physical Education and Sport, 03150 Kyiv, Ukraine; 3 Department of Biomedical Sciences, University of Lausanne, 1005 Lausanne, Switzerland; 4 Angiology Division, Heart and Vessel Department, Lausanne University Hospital (CHUV), 1011 Lausanne, Switzerland; lucia.mazzolai@chuv.ch

**Keywords:** exercise intensity, normobaric hypoxia, gene expression, metabolic pathways, mice, skeletal muscle

## Abstract

The muscle molecular adaptations to different exercise intensities in combination with hypoxia are not well understood. This study investigated the effect of low- and supramaximal-intensity hypoxic training on muscle metabolic gene expression in mice. C57BL/6 mice were divided into two groups: sedentary and training. Training consisted of 4 weeks at low or supramaximal intensity, either in normoxia or hypoxia (FiO_2_ = 0.13). The expression levels of genes involved in the hypoxia signaling pathway (*Hif1a* and *Vegfa*), the metabolism of glucose (*Gys1*, *Glut4*, *Hk2*, *Pfk*, and *Pkm1*), lactate (*Ldha*, *Mct1*, *Mct4*, *Pdh*, and *Pdk4*) and lipid (*Cd36*, *Fabp3*, *Ucp2*, *Hsl*, and *Mcad*), and mitochondrial energy metabolism and biogenesis (*mtNd1*, *mtNd6*, *CytC*, *CytB*, *Pgc1a*, *Pgc1β*, *Nrf1*, *Tfam*, and *Cs*) were determined in the gastrocnemius muscle. No physical performance improvement was observed between groups. In normoxia, supramaximal intensity training caused upregulation of major genes involved in the transport of glucose and lactate, fatty acid oxidation, and mitochondrial biogenesis, while low intensity training had a minor effect. The exposure to hypoxia changed the expression of some genes in the sedentary mice but had a moderate effect in trained mice compared to respective normoxic mice. In hypoxic groups, low-intensity training increased the mRNA levels of *Mcad* and *Cs*, while supramaximal intensity training decreased the mRNA levels of *Mct1* and *Mct4*. The results indicate that hypoxic training, regardless of exercise intensity, has a moderate effect on muscle metabolic gene expression in healthy mice.

## 1. Introduction

Exercise training is an effective means for the achievement of health-promoting effects, including changes in skeletal muscle metabolism. Growing evidence suggests that both low and high exercise intensities lead to improvements in skeletal muscle metabolic adaptations [1,2,3,4,5]. Similarly to moderate-intensity continuous training, both high-intensity interval training (HIIT) and supramaximal interval training (SIT, or sprint) may induce increased aerobic capacity (VO_2max_) and muscle mitochondrial content [6]. The intensity of SIT (“all-out” efforts) is not only time-efficient to improve muscle glycolytic capacity, but it may also induce a shift of skeletal muscle fibers towards a more oxidative phenotype [7].

Growing evidence shows that combining high-intensity exercise and hypoxic stimuli may lead to an additional benefit to physical performance when compared to a similar exercise in normoxia [8]. Moreover, by using moderate hypoxia itself, one may trigger the up-regulation of a number of genes involved in energy metabolism [5].

Despite the hypoxia-inducible factor (HIF) being considered the primary mediator of cellular hypoxia, previous analyses performed on skeletal muscle tissue indicate that HIF-1 protein expression is only slightly affected by passive exposure to moderate hypoxia [9]. Furthermore, the assertion that chronic hypoxia itself would promote angiogenesis or upregulation of oxidative enzymes is disputed [9]. Moreover, recent evidence shows that hypoxia can regulate genes involved in glucose metabolism at rest and those involved in myoblast differentiation, fusion, and muscle contraction after exercise [10]. The influence of exercise intensity in hypoxia may provide a potential benefit of combining high-intensity and severe hypoxia to improve O_2_ transport and skeletal muscle metabolism [11]. Indeed, combining supramaximal intensity and hypoxia (i.e., repeated sprint training in hypoxia, RSH) led to larger improvements in both anaerobic and aerobic performance when compared to the same exercise in normoxia [12,13]. Overall, RSH may improve performance through a decrease in O_2_ availability, leading to an increase in HIFs and target genes at the cellular level of skeletal muscles. Among these adaptations, RSH upregulated some genes involved in glucose metabolism and mitochondrial biogenesis, depending on the training protocol [12,14,15]. Previous studies have also examined the effects of hypoxic exercise training on muscle mitochondrial turnover and metabolism in mice [16,17]. However, there are few studies comparing the effects of different exercise intensities, superimposed with hypoxia, on molecular responses in skeletal muscle. The goal of this study was to investigate the effects of low and supramaximal exercise intensities in hypoxia and normoxia on the expression of genes related to skeletal muscle energy metabolic pathways in C57BL/6 mice.

## 2. Materials and Methods

### 2.1. Animal and Experimental Protocol

**Figure 1:**
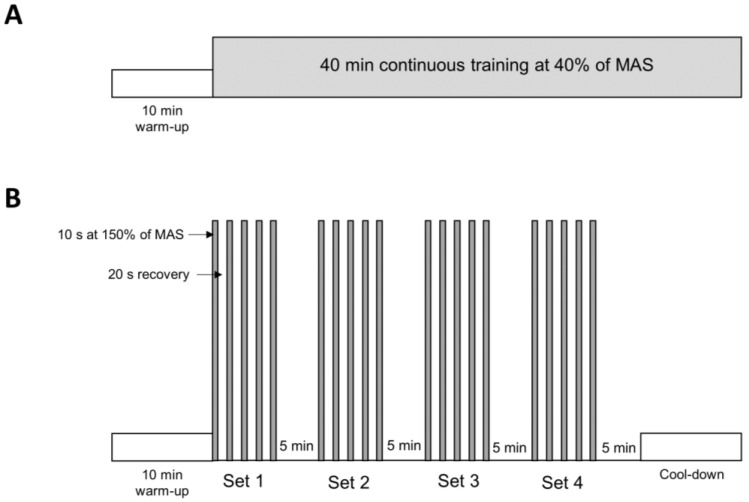
Schematic illustration of the two training protocols. LIT-trained mice ran 40 min at 40% of maximal aerobic speed (MAS) (**A**). HIIT-trained mice ran 4 sets composed of 10 s sprints at 150% of MAS interspersed by 20 s rest, and a 5 min pause between the sets (**B**). Each training session was preceded by a 10 min warm-up and ended with a cool-down period.

An internal animal experimentation committee as well as the Veterinary Office of the Canton de Vaud approved all animal experiments (authorization VD3224). The Swiss animal experimentation laws and guidelines were followed throughout the described experiments. Thirty-six 8-week-old male C57BL/6J healthy mice (Charles River Laboratories, L’Arbresle, France) were randomly divided into six groups (*n* = 6/group): sedentary (SED), low-intensity training (LIT), and supramaximal intensity training (SIT). Each group was exposed to either normoxia (fraction of inspired O_2_ of room air, F_i_O_2_ = 0.21) or hypoxia (F_i_O_2_ = 0.13) according to a previously published protocol by our group [18]. Mice were housed in a ventilated, temperature- and humidity-controlled environment under a 12-h light/dark cycle. All groups had *ad libitum* access to a standard diet and water. The two intensities of exercise were determined based on the maximal aerobic speed (MAS). MAS was established using an incremental test-to-exhaustion (in normoxia), as previously described [18,19]. Mice assigned to LIT (2 subgroups) ran continuously for 40 min at 40% of their MAS (Figure 1). The SIT mice (2 subgroups) ran 4 sets of 5 × 10 s sprints at 150% of MAS, with 20 s of passive recovery between each sprint (Figure 1). The interset rest was 5 min of passive recovery (Figure 1). The training intervention lasted 4 weeks, with 3 sessions/week for all groups. The mice ran on a rodent treadmill (Panlab LE-8710, Bioseb, Vitrolles, France). To match the total workload of the LIT mice, the SIT group underwent a cool-down period after each training session. The treadmill was placed in a chamber under normoxic or hypoxic conditions. All mice in the hypoxic groups were exposed to the same dose of moderate normobaric hypoxia (F_i_O_2_ of 0.13 for 50 min). The incremental test was repeated at the end of the study to determine maximal running distance, as previously described [20]. The body weight of the mice was measured weekly during the experimental period.

### 2.2. Quantitative Real-Time Polymerase Chain Reaction

Twenty-four hours after the final incremental test, the mice were euthanized by cervical dislocation after anesthesia via isoflurane inhalation. Gastrocnemius muscle samples were collected, snap-frozen in liquid nitrogen, and stored at −80 °C. About 30 mg of muscle tissue (including both white and red portions) were placed in lysis buffer, disrupted, and then homogenized utilizing a Tissue Lyser LT (Qiagen, Basel, Switzerland). According to the manufacturer’s protocols, total RNA was isolated using an RNeasy Fibrous Tissue Mini Kit (Qiagen, Basel, Switzerland) and reverse transcribed into cDNA using a PrimeScript RT Reagent Kit with a gDNA eraser (TaKaRa Bio Inc., Shiga, Japan). The real-time PCR started at 50 °C for 1 min, then 95 °C for 1 min, and was followed by 40 thermal cycles at 95 °C for 1 s, 60 °C for 1 min, 95 °C for 15 s, 60° for 1 min, and 95 °C for 15 s. A qRT-PCR test was performed in triplicate for each sample using a ViiA7 Real-Time PCR detection system (Applied Biosystems, Waltham, MA, USA). All primer sequences are listed in Supplementary Table S1. For each sample, the cycle threshold (Ct) of each target gene was normalized to the Ct of the housekeeping gene *18s* to determine ∆Ct. The *18s* gene was selected as the housekeeping gene because it showed invariant expression across our experimental conditions. To relatively quantify gene expression, the 2-∆∆CT method was used.

### 2.3. Statistical Analysis

A two-way ANOVA was performed to analyze the main effect of O_2_ level (normoxia vs. hypoxia) and intervention (SED vs. LIT vs. SIT), as well as the interaction between these 2 factors on gene expression. Data normality was verified by using the Shapiro–Wilk test. The Bonferroni test was applied for multiple comparisons among the experimental groups after a significant interaction. Statistical analysis was performed using GraphPad Prism 9 (version 9.4.1) (GraphPad Software, Inc, San Diego, CA, USA). All data are presented as the mean ± standard deviation (SD). Results were considered statistically significant at *p* < 0.05.

## 3. Results

### 3.1. Effect on Performance and Body Weight

**Figure 2:**
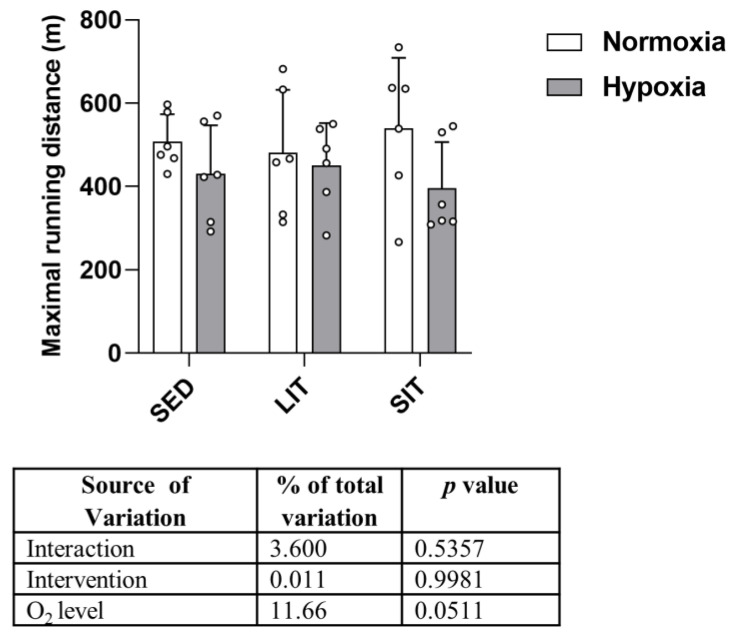
Treadmill physical performance of normoxic and hypoxic SED, LIT, and SIT mice. Treadmill test was performed by increasing speed by 2 cm/s every 3 min until exhaustion.

No significant differences in physical performance were observed between groups (Figure 2). The maximum running distance at the end of the study was 508 ± 66 m in SED in normoxia vs. 431 ± 116 m in SED in hypoxia vs. 481 ± 151 m in LIT in normoxia vs. 451 ± 101 in LIT in hypoxia vs. 540 ±169 m in SIT in normoxia vs. 396 ± 111 m in SIT in hypoxia (Figure 2). Over the experimental protocol, body weight was significantly reduced in LIT and SIT compared to SED in normoxic conditions, while no significant differences were found between the hypoxic groups (Supplementary Figure S1).

### 3.2. Hypoxia Signaling Pathway

**Figure 3:**
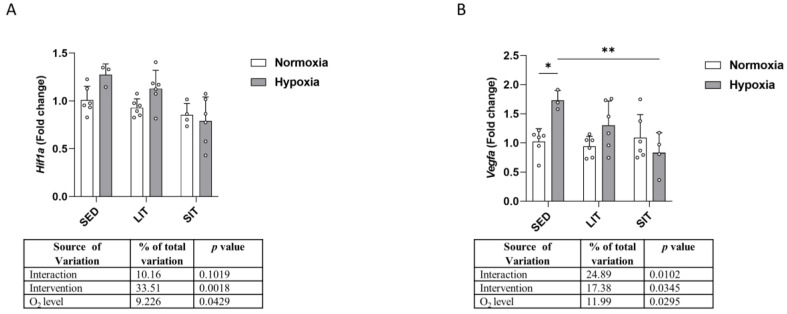
The qRT-PCR analysis of *Hif1a* (**A**) and one of its target genes, *Vegfa* (**B**). Gene expression levels are normalized to the housekeeping gene (*18s*) and relative to SED in normoxia. Asterisks represent significance as determined by a two-way ANOVA (* *p* < 0.05; ** *p* < 0.01). Gene abbreviations are: *Hif1a* (hypoxia-inducible factor-1α); *Vegfa* (vascular endothelial growth factor A).

As shown in Figure 3A, there was a statistically significant main effect of O_2_ level on *Hif1a* expression, with mRNA levels being increased in hypoxic SED and LIT groups, when compared to their respective normoxic groups. Expression of the *Hif1a* target gene vascular endothelial growth factor A (*Vegfa*) was higher in hypoxic SED than in normoxic SED (Figure 3B). *Vegfa* mRNA expression was lower in SIT compared to SED in hypoxic conditions (Figure 3B).

### 3.3. Glucose Metabolism

**Figure 4:**
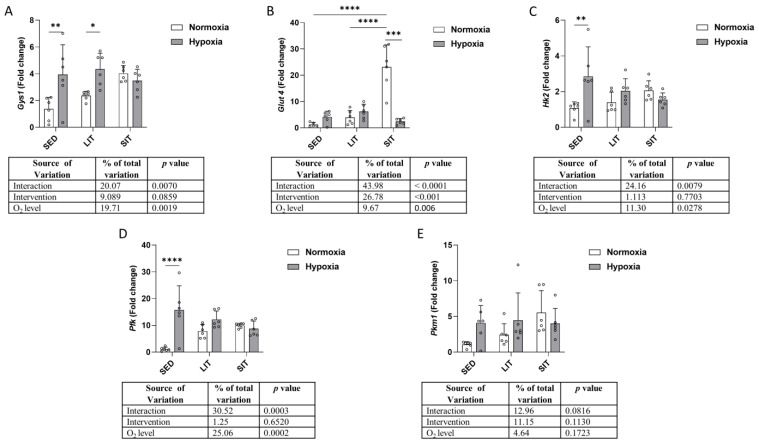
The qRT-PCR analysis of the indicated genes involved in glycogen synthesis (**A**), glucose transport (**B**), and glycolysis (**C**–**E**). Gene expression levels are normalized to the housekeeping gene (*18s*), and relative to SED in normoxia. Asterisks represent significance as determined by a two-way ANOVA (* *p* < 0.05; ** *p* < 0.01; *** *p* < 0.001; **** *p* < 0.0001). Gene abbreviations are: *Gys1* (glycogen synthase 1); *Glut4* (solute carrier family 2 member 4); *Hk2* (hexokinase 2); *Pfk* (phosphofructokinase); *Pkm1* (pyruvate kinase muscle 1).

The expression levels of glycogen synthase 1 (*Gys1*), solute carrier family 2 member 4 (*Glut4*); hexokinase 2 (*Hk2*); phosphofructokinase (*Pfk*); and pyruvate kinase muscle 1 (*Pkm1*) are shown in Figure 4. *Gys1* expression was higher in hypoxic SED and LIT but not in SIT, compared to their respective normoxic groups (Figure 4A). The expression of *Glut4* was higher in SIT compared to LIT and SED in normoxia, while no significant differences were found between hypoxic groups (Figure 4B). *Glut4* expression was lower in SIT under hypoxic compared to normoxic conditions (Figure 4B). Hypoxia upregulated *Hk2* and *Pfk* expression in SED but not in LIT and SIT groups (Figure 4C,D). *Pkm1* expression was not different between groups (Figure 4E).

### 3.4. Lactate Metabolism

**Figure 5:**
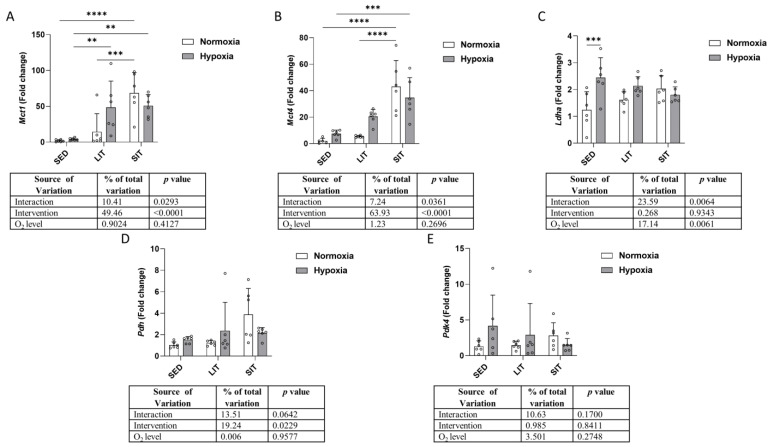
The qRT-PCR analysis of the indicated genes related to lactate transport (**A**,**B**) and production (**C**–**E**). Gene expression levels are normalized to the housekeeping gene (*18s*) and relative to SED in normoxia. Asterisks represent significance as determined by a two-way ANOVA (** *p* < 0.01; *** *p* < 0.001; **** *p* < 0.0001). Gene abbreviations are: *Mct1* (monocarboxylate transporter 1); *Mct4* (monocarboxylate transporter 4); *Ldha* (lactate dehydrogenase A); *Pdh* (pyruvate dehydrogenase); *Pdk4* (pyruvate dehydrogenase kinase 4).

Figure 5 displays changes in mRNA expression levels of monocarboxylate transporter 1 (*Mct1*), monocarboxylate transporter 4 (*Mct4*), lactate dehydrogenase A (*Ldha*), pyruvate dehydrogenase (*Pdh*), and pyruvate dehydrogenase kinase 4 (*Pdk4*). *Mct1* mRNA expression was upregulated in SIT compared to SED and LIT, while in hypoxic groups, its expression was upregulated both in SIT and LIT as compared to SED (Figure 5A). Regarding *Mct4*, its expression was also higher in SIT than in SED and LIT in normoxic groups, while a higher expression could be detected only in SIT compared to SED in hypoxic groups (Figure 5B). Hypoxia significantly increased *Ldha* expression in SED but not in LIT or SIT (Figure 5C). In normoxic groups (Figure 5D), either exercise intervention had a significant effect on *Pdh* expression, with increasing mRNA levels in exercised groups compared to SED. No significant difference was found in *Pdk4* expression between groups (Figure 5E).

### 3.5. Mitochondrial Biogenesis and Krebs Cycle

**Figure 6:**
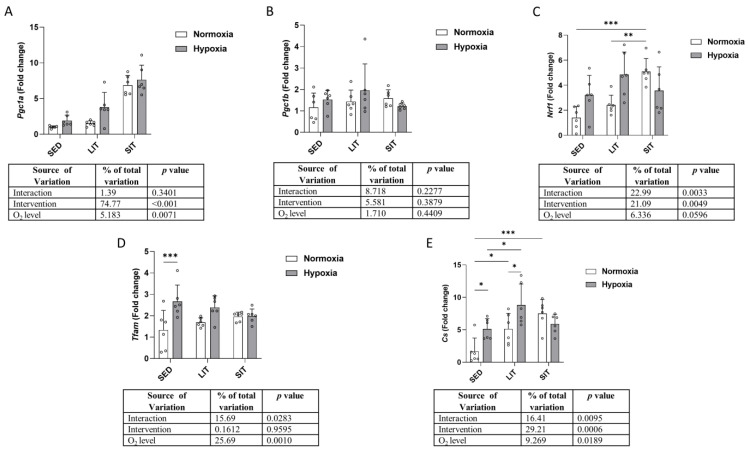
The qRT-PCR analysis of the indicated genes responsible for mitochondrial biogenesis (**A**–**D**) and Krebs cycle (**E**). Gene expression levels are normalized to the housekeeping gene (*18s*) and relative to SED in normoxia. Asterisks represent significance as determined by a two-way ANOVA (* *p* < 0.05; ** *p* < 0.01; *** *p* < 0.001). Gene abbreviations are: *Pgc1a* (peroxisome proliferator-activated receptor gamma coactivator 1-alpha); *Pgc1b* (peroxisome proliferator-activated receptor gamma coactivator 1-beta); *Nrf1* (nuclear respiratory factor 1); *Tfam* (mitochondrial transcription factor A); *Cs* (citrate synthase).

As shown in Figure 6A,B, both exercise intervention and O_2_ level had an effect on peroxisome proliferator-activated receptor gamma coactivator 1-alpha (*Pgc1a*) expression but not on peroxisome proliferator-activated receptor gamma coactivator 1-beta (*Pgc1b*) expression. Nuclear respiratory factor 1 (*Nrf1*) mRNA expression was higher in SIT than in SED and LIT only in normoxic groups (Figure 6C). Hypoxia induced higher mitochondrial transcription factor A (*Tfam*) expression in SED but not in LIT or SIT (Figure 6D). As shown in Figure 6E, citrate synthase (*Cs*) expression was higher in hypoxic groups than in normoxic groups in SED and LIT, but not in SIT. Between normoxic groups, *Cs* expression was higher in LIT and SIT than in SED, while this difference was only observed in LIT in the hypoxic groups (Figure 6E).

### 3.6. Mitochondrial Respiratory Chain Complex

**Figure 7:**
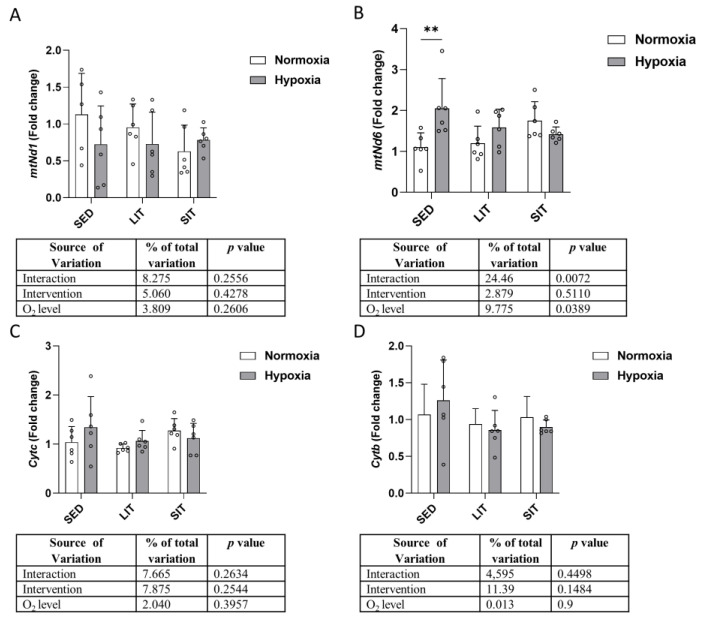
The qRT-PCR analysis of the indicated genes encoding mitochondrial respiratory chain complex (**A**–**D**). Gene expression levels are normalized to the housekeeping gene (*18s*) and relative to SED in normoxia. Asterisks represent significance as determined by a two-way ANOVA (** *p* < 0.01). Gene abbreviations are: *mtNd1* (mitochondrial NADH dehydrogenase 1); *mtNd6* (mitochondrial NADH dehydrogenase 6); *Cytc* (mitochondrial cytochrome C); *Cytb* (mitochondrial cytochrome B).

No significant differences were found in the mRNA expression of mitochondrial NADH dehydrogenase 1 (*mtNd1*) (Figure 7A), mitochondrial cytochrome C (*Cytc*) (Figure 7C), and mitochondrial cytochrome B (*Cytb*) (Figure 7D) between groups. Mitochondrial NADH dehydrogenase 6 (*mtNd6*) expression was higher in SED exposed to hypoxia than normoxia (Figure 7B).

### 3.7. Fatty Acid β-Oxidation

**Figure 8:**
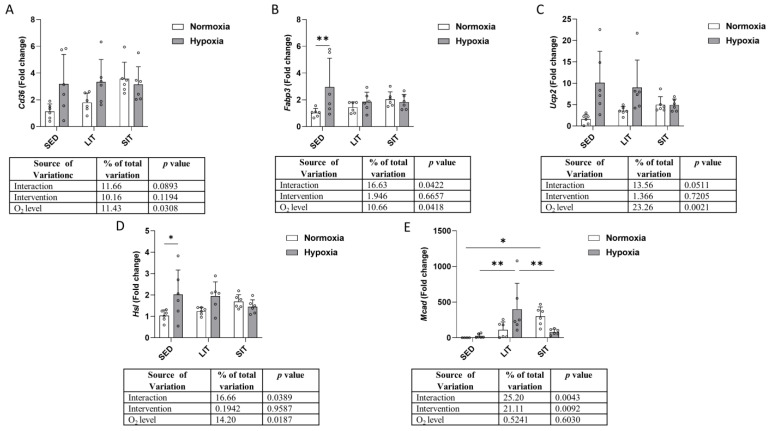
Gene expression analysis of the indicated gene involved in fatty acid uptake (**A**), transport (**B**), and fatty acid β-oxidation (**C**–**E**). Gene expression levels are normalized to the housekeeping gene (*18s*) and relative to SED in normoxia. Asterisks represent significance as determined by a two-way ANOVA (* *p* < 0.05; ** *p* < 0.01). Gene abbreviations are: *Cd36* (fatty acid translocase cluster of differentiation); *Fabp3* (fatty acid binding protein 3); *Ucp2* (uncoupling protein 2); *Hsl* (hormone-sensitive lipase); *Mcad* (medium-chain acyl-CoA dehydrogenase).

We measured the mRNA expression of five genes implicated in fatty acid uptake, transport, and fatty acid β-oxidation (fatty acid translocase cluster of differentiation, *Cd36*; fatty acid binding protein 3, *Fabp3*; uncoupling protein 2, *Ucp2*; hormone-sensitive lipase, *Hsl*; medium-chain acyl-CoA dehydrogenase, *Mcad*) (Figure 8). There was a significant main effect of O_2_ level on *Cd36* and *Ucp2* expression, with mRNA levels increasing in hypoxic SED and LIT compared to their respective normoxic groups (Figure 8A,C). *Fabp3* and *Hsl* expression were higher in hypoxic than normoxic groups, only in SED (Figure 8B,D). As shown in Figure 8E, *Mcad* expression was higher in SIT compared to SED in normoxic groups. In hypoxic groups, its expression was higher in LIT than in SED and SIT (Figure 8E). Moreover, *Mcad* expression was lower in hypoxic SIT than in normoxic SIT (Figure 8E).

## 4. Discussion

The effects of two different types of exercise training (i.e., at low and high intensities) in hypoxia or normoxia on the endurance performance of C57BL/6 mice and the expression of genes involved in energy metabolic pathways in the gastrocnemius muscle are summarized in four points, as follows:

(1) None of the 4-week training protocols, either in normoxia or hypoxia, led to an increase in exercise performance, suggesting an insufficient training load.
(2) Supramaximal exercise training in normoxia caused upregulation of some genes involved in glucose and lactate transport as well as some genes responsible for mitochondrial biogenesis and fatty acid oxidation.
(3) The exposure to hypoxia induced a higher expression of genes involved in glucose metabolism and mitochondrial biogenesis, mainly in the sedentary mice.
(4) Exercise training performed in hypoxia had a moderate effect on these transcriptional adaptations.

In the present study, no differences in maximal running distance were reported between groups. Studies conducted on mice often display heterogeneous results when looking into the impact of exercise training on performance. These discrepancies are due to specificities of exercise protocol, exercise performance test type, outcome variables, and mouse genetic background [21,22,23]. The result of the present performance test is in line with previous studies that did not report performance improvement in C57BL/6 mice after 4 weeks of exercise training in normoxia [22,24].

GLUT4 is a critically important protein that provides glucose transport in skeletal muscle cells and carries out muscle glucose uptake during contraction/exercise [25]. It was previously shown that GLUT4 mRNA expression was significantly increased by both moderate- and high-intensity exercise in the gastrocnemius muscle of rodents [26]. Interestingly, we reported upregulation of the GLUT4 mRNA level with SIT. This suggests that SIT can also be an efficient stimulus for the transcriptional regulation of the GLUT4 gene in skeletal muscle.

Previous studies reported changes in the mRNA and protein levels of GLUT4 in skeletal muscle in response to hypobaric hypoxia [9,27], as well as in athletes who performed bouts of repeated sprint exercise in hypoxic conditions [14]. Our findings contradict these later results and suggest that hypoxia superimposed on supramaximal training may blunt GLUT4 gene expression.

Hypoxia led to a significant increase in GYS1 gene expression, both in sedentary and low-intensity exercise training groups. These data are consistent with the results of previous studies that established that GYS1 is a hypoxia-regulated HIF target gene [28]. Low-intensity training itself (treadmill walking and/or jogging) did not alter the total activity of glycogen synthase in human vastus lateralis muscle [29]. Twelve weeks of endurance exercise training (75–90% of maximal heart rate) increased the expression of GYS1 in all skeletal muscle fiber types, which probably contributed to the increased post-training glycogen content in humans [30].

Our study indicates a significant increase in the level of HK2 gene expression in moderate hypoxic conditions. HK2 is an HIF-1 target gene and, during the early stages of exercise, a key determinant of muscle glucose uptake [31]. It is widely considered that hypoxia induces HK2 gene expression in different tissues [32]. Previous studies revealed that the level of HK2 mRNA in human muscle after up to 8 h of exposure to a high-altitude (hypobaric) hypoxia was not changed [9], while an increase in protein expression was observed in a hypobaric chamber simulating an ascent to the summit of Mount Everest [33] and after endurance training at high altitude [34]. Overall, it appears that there is a large body of evidence indicating that hypoxia induces the overexpression of HK2. The effects of hypoxia on PFK gene expression are debated, with previous studies reporting either no change [9,35] or blunted expression [12] with exposure to hypoxia. In contrast, we observed a significant increase in the level of PFK gene expression under moderate hypoxia and normobaric conditions. The reasons for such a discrepancy remain unclear.

It is well known that lactate production and transport are closely related to exercise intensity. Our findings suggest that only supramaximal exercise training led to activation of lactate metabolism genes. Indeed, our results show that SIT caused a significant increase in MCT1 and MCT4 gene expression, confirming that genes responsible for lactate transport were activated with such a training modality. Our results are consistent with a large body of research demonstrating increased MCT1 and MCT4 expression with supramaximal exercise training [36,37]. This is explained by considering that SIT is performed mainly by fast-twitch fibers. MCT1 is predominantly expressed in slow-twitch muscle fibers, where it facilitates the uptake of lactate into working skeletal muscles, while MCT4 is expressed in fast-twitch fibers, where it mediates lactate efflux [38]. The additional influence of hypoxia remains unclear; for example, MCT1 and MCT4 protein content did not significantly differ before and after 3 weeks of intermittent hypoxic training in trained athletes (2 sets of 3 repetitions each, 2 min in duration, and at an intensity of 100% peak power output) [39]. Hence, the addition of the hypoxic stimulus after a 3-week training period was ineffective on MCT expression [39]. Overall, our data suggest that supramaximal intensity training triggers greater activation of MCT1 and MCT4 genes than exposure to hypoxia itself.

Mitochondrial biogenesis is an important adaptation of skeletal muscle to exercise training. Most of the research concerns low-intensity exercise training that exhibits enhanced expression of PGC-1s [40,41]. Several studies claim that HIIT activates mitochondrial biogenesis-related signaling pathways linked to PGC-1α, which is a key regulator of mitochondrial biogenesis and can activate TFAM and NRFs [42,43]. HIIT appears to be a potent training stimulus, as effective as 40–60 min of continuous moderate-intensity training per session for increasing mitochondrial content [44,45,46] through several modifications in signaling cascades, including the protein phosphorylation of ACC and p38MAPK and the mRNA expression of PGC-1α [47,48].

In our study, TFAM was upregulated by hypoxia only in the SED group but not in the exercising groups, and no differences were reported in the expression of PGC-1α and PGC-1β. The PGC-1α mRNA level was increased after 6 weeks of HIIT in human muscle [49], but not after 9 days of endurance training [50]. Overall, these data suggest that mitochondrial biogenesis requires a longer training period than the 4 weeks of the present study.

One interesting result of our study is the significant activation of the NRF1 gene only in the normoxic SIT group. It is well known that NRF1 is a pivotal coordinator of the mitochondrial biogenesis pathway and has been reported to be associated with the physiological functions of skeletal muscle [38]. NRF1 up-regulates the expression of TFAM, which is subsequently transported into the mitochondria, as well as nuclear genes encoding mitochondrial proteins [51]. There are certain contradictions in the understanding of the functioning of this transcription factor. A number of scientists believe that PGC-1α coactivates NRF1 targets [52], while others believe that it can be activated independently of *PGC-1*α [53]. Our data are in line with previous observations that, in rat skeletal muscles, NRF1-dependent mitochondrial biogenesis happens prior to up-regulation of PGC-1α levels in response to exercise [54].

In the present study, both types of exercise training caused a significant increase in CS gene expression compared to the sedentary group in normoxia. Hypoxia also triggered CS mRNA expression, except when combined with SIT. CS activity is a reliable biomarker of mitochondrial content [55] and correlates with aerobic capacity, indicating a possible long-term effect of training [56].

Overall, our data support the beneficial effect of hypoxia on CS and TFAM gene expression, as previously shown; e.g., intermittent normobaric hypoxia exposure increased expression of TFAM, especially when combined with aerobic exercise [57]. TFAM is one of the key regulators of mitochondrial DNA replication and gene transcription and enhances skeletal muscle energy metabolism due to increased mitochondrial oxidative capacity [58].

It was reported that SIT and moderate-intensity exercise may increase mitochondrial content to a similar extent, while exercise intensity mainly influences metabolic signals for mitochondrial biogenesis [6]. Here, we showed that exercise intensity, either in normoxia or hypoxia, had no effect on expression of four genes related to the mitochondrial respiratory chain complex. This can be explained by the short training intervention and insufficient time for releasing factors mediating exercise-induced mitochondrial adaptations, which may be associated with a lack of training-induced changes in gene expression of mitochondrial biogenesis transcriptional factors such as PGC-1α and PGC1-1β. It is in accordance with some previous studies demonstrating no significant changes in ND1, ND6, CYTB, and CYTC mRNA levels with different protocols of exercise training [44,59].

Endurance exercise training improves fatty acid oxidation due to increased expression of CD36 and CPT I [50]. For example, 4 weeks of exercise training stimulated mRNA expression of genes involved in fatty acid metabolism, such as CD36, FABP3, HSL, and UCP2, in mice [59]. It is generally considered that the increase in CD36 gene expression is a mechanism whereby fatty acid uptake and oxidation are increased in trained skeletal muscle of humans. Surprisingly, we did not find CD36 up-regulation either at low- or supramaximal-intensity training. One may speculate that the training load was too small to induce such changes. Interestingly, we report increased mRNA levels of CD36 and HSL with hypoxia in the sedentary mice.

HSL is a major determinant of fatty acid mobilization in skeletal muscle fibers and displays triacylglycerol hydrolysis activity. The expression in skeletal muscle is correlated with fiber type, being higher in oxidative fibers than in glycolytic fibers [60]. It was previously established that different types of exercise training (including HIIT) lead to increased lipolysis due to increased HSL gene expression and protein concentration in adipocytes and skeletal muscle, both in humans and in animals [61,62,63,64,65]. The same effect is exerted on the expression of this gene by hypoxia; e.g., intermittent hypoxia increased HSL-mediated lipolysis in mice [66,67]. Since HSL is under the hormonal control of catecholamines and natriuretic peptides, one may hypothesize that the increased HSL gene expression is due to sympathetic activation in response to hypoxia exposure [68].

The observed higher FABP3 mRNA level in the hypoxic SED group compared to the normoxic group is in line with a previous observation that FABP3 is activated by hypoxia in a HIF-1α-dependent manner [69]. FABP3 is mainly expressed in cardiomyocytes and skeletal muscle cells and facilitates the cytoplasmic transport of fatty acids between intracellular membranes.

## 5. Conclusions

In conclusion, none of the 4-week training protocols in either normoxia or hypoxia led to an increase in exercise performance in C57BL/6 mice. However, supramaximal exercise training in normoxia caused upregulation of key genes involved in glucose and lactate transport as well as in mitochondrial biogenesis and fatty acid oxidation. The exposure to hypoxia induced a higher expression of genes involved in glucose metabolism and mitochondrial biogenesis, mainly in the sedentary mice, but exercise training performed in hypoxia had a moderate effect on the transcriptional adaptations. Our study provides new insights into the molecular adaptations of skeletal muscle to different exercise regimens in normoxic and hypoxic conditions.

## Data Availability

Data is not publicly available due to privacy or ethical restrictions.

## Supplementary Materials

The following supporting information can be downloaded at: https://www.mdpi.com/article/10.3390/metabo13101103/s1, Table S1: Primer sequences used for qRT-PCR; Figure S1: Body weight gain in normoxic and hypoxic SED, LIT and SIT mice.
