# Supplementary material for: Moderate Effects of Hypoxic Training at Low and Supramaximal Intensities on Skeletal Muscle Metabolic Gene Expression in Mice
Source: Metabolites. 2023 Oct 21;13(10):1103. doi: 10.3390/metabo13101103 (PMC10609052; doi:10.3390/metabo13101103)
Supplement: Supplementary file 1 [file metabolites-13-01103-s001.zip › metabolites-2628098-supplementary.pdf]

Supplementary Table S1. Primer sequences used for qRT-PCR

Gene	Forward sequence (5′-3′)	Reverse sequence (5′-3′)
18s	ACTTTTGGGGCCTTCGTGTC	GCCCAGAGACTCATTTCTTCTTG
Hif1a	TCAAGTCAGCAACGTGGAAG	TATCGAGGCTGTGTCGACTG
Vegfa	GTACCTCCACCATGCCAAGT	GCATTCACATCTGCTGTGCT
Gys1	GAGAACGCAGTGCTTTTCGA	TCATCCCCTGTCACCTTCG
Glut4	CTCTCAGGCATCAATGCTGTTTTCTA	CGAGACCAACGTGAAGACCGTATT
Hk2	GCTAGGAGCTACCACACACCCT	ACTCGCCATGTTCTGTCCCATCC
Pfk	GGAGTGCGTGCAGGTGACCAAA	ATCACGGCCACTGTGTGCAACC
Pkm1	CAG CCA AAG GGG ACT ATC CT	GAG GCT CGC ACA AGT TCT T
Mct1	TTGTCTGTCTGGTTGCGGCTTGATCG	GCCCAAGACCTCCAATAACACCAATGC
Mct4	GCCACCTCAACGCCTGCTA	TGTCGGGTACACCCATATCCTTA
Ldha	TGCCTACGAGGTGATCAAGCT	GCACCCGCCTAAGGTTCTTC
Pdh	GAAGGCCCTGCATTCAACTTC	ATAGGGACATCAGCACCAGTG A

Pdk4	TACTCCACTGCTCCAACACCTG	AGCCATAACCAAAACCAGCCAAAG
Pgc1a	ACTATGAATCAAGCCACTACAGAC	TTCATCCCTCTTGAGCCTTTCG
Pgc1b	GAGGAGTCCCTTCCTTCATC	TCCTCGAAGGTTAAGGCTGA
Nrf1	GCACCTTTGGAGAATGTGGT	CTGAGCCTGGGTCATTTTGT
Tfam	CCAAAAAGACCTCGTTCAGC	CTTCAGCCATCTGCTCTTCC
Cs	GGACAATTTTCCAACCAATCTGC	TCGGTTCATTCCCCTCTGCATA
mtNd1	GCACCTACCCTATCACTCACA	GTTTGGGCTACGGCTCG
mtNd6	TACCCGCAAACAAAGATCACC	ATGTTGGAAGGAGGGATTGGG
Cytc	CCAAATCTCCACGTTCTGTT	GTCTGCCCTTTCTCCCTTCT
Cytb	ACGCAAACGGAGCCTCAATA	CCTCATGGAAGGACGTAGCC
Cd36	ATGGGCTGTGATCGGAACTG	GTCTTCCCAATAAGCATGTCTCC
Fapb3	ACCTGGAAGCTAGTGGACAG	TGATGGTAGTAGGCTTGGTCAT
Ucp2	GCTCAAGAAACTAATCACAGCCA	GGTGGGCTTATTCTACCATTG
Hsl	TTCCCTGTTGATGTGGTCAA	CAGTGACCTGCGCTGTGGTA

Mcad	GATCGCAATGGGTGCTTTTGATAGAA	AGCTGATTGGCAATGTCTCCAGCAAA

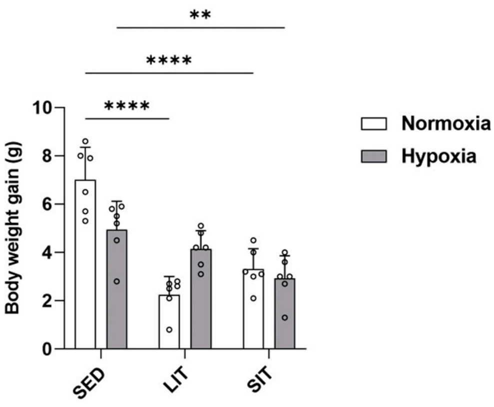

Source of Variation	% of total variation	*p* value
Interaction	20.22	0.0001
Intervention	54.25	<0.0001
O_2 level	0.2437	0.5947

Supplementary Figure S1. Body weight gain in normoxic and hypoxic SED, LIT and SIT mice. Asterisks represent significance as determined by a two-way ANOVA (** p < 0.01; **** p < 0.0001).
